# Comparison of the Curative Effect of Percutaneous Reduction with Plastic Calcaneal Forceps Combined with Medial External Fixation in the Treatment of Intra‐Articular Calcaneal Fractures

**DOI:** 10.1111/os.13118

**Published:** 2021-11-12

**Authors:** Jianchuan Wang, Song Qin, Tienan Wang, Jibin Liu, Zongpu Wang

**Affiliations:** ^1^ Department of Orthopedics Affiliated Zhongshan Hospital of Dalian University Dalian China

**Keywords:** Calcaneus, Intra‐articular fractures, Minimally invasive, Percutaneous fixation, Tarsal sinus approach

## Abstract

**Objective:**

To compare the clinical efficacy of percutaneous minimally invasive reduction combined with external fixation and a tarsal sinus approach to treat Sanders type II and III intra‐articular calcaneal fractures.

**Methods:**

The clinical data of 64 patients with Sanders type II and III calcaneal fractures admitted to our hospital from January 2010 to January 2016 were retrospectively analyzed; data includedage, sex, body mass index. According to the surgical method, they were divided into the percutaneous minimally invasive reduction with internal and external fixation group (30 cases) and the tarsal sinus approach group (34 cases).The two groups of patients were compared in terms of the time tosurgery, length of hospital stay, intraoperative blood loss, operative duration, complications, radiographic features, including the heel bone length, width, height, Bohlerangle, Gissane angle, and calcaneal varus angle, and clinical efficacy indicators, including the American Orthopedic Foot and Ankle Society (AOFAS) score, the visual analog scale (VAS) pain score, health survey profile (SF‐36) score and Maryland ankle function score.

**Results:**

Patients in both groups were followed up for 12 to 50 months, with an average of 24.8 months.Bony union was achieved in all cases. The time to surgery, length of hospitalstay, intraoperative blood loss and incidence of incision‐related complications were significantly lower in the percutaneous minimally invasive medial external fixation group than in the tarsal sinus group (*P* < 0.01). At the last follow‐up, the calcaneal length, width, and height, Bohler angle, Gissane angle, and varus angle were significantly increased in both groups (*P* < 0.01), the calcaneal width was significantly lower after than before surgery (*P* < 0.01), and there were no statistically significant differences between the two groups (*P* > 0.05). As measures of clinical efficacy, the AOFAS, VAS, SF‐36 and Maryland scores were 85.28 ± 8.21, 0.84 ± 1.21, 82.95 ± 3.25 and 83.56 ± 3.32, respectively, at the last follow‐up in the percutaneous minimally invasive medial external fixation group and 83.32 ± 7.69, 1.85 ± 1.32, 80.71 ± 5.42, and 81.85 ± 2.41 in the tarsal sinus group, respectively, with no significant differences between the two groups (*P* > 0.05).

**Conclusion:**

Under the condition of a good command of surgical indications and surgical skills, the use of plastic calcaneal forceps for percutaneous minimally invasive reduction combined with medial external fixation for the treatment of Sanders type II and III intra‐articular calcaneal fractures can achieve similar clinical effects as the tarsal sinus approach. However, the use of plastic calcaneal forceps for percutaneous minimally invasive reduction combined with internal and external fixation has advantages, such as fewer complications, less bloodloss, and a shorter operation, and thus has good safety and is worthy of clinical promotion.

## Graphical abstract

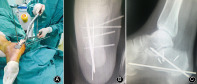

The anatomical structure of the calcaneus was restored by applying traction and extrusion with calcaneus‐shaping forceps. On axial X‐ray, 4 nails were arranged from the subtalar joint to the calcaneal tuberosity in different planes to provide fracture surface support. Two Kirschner wires drilled from the posterior tuberosity were fixed in the talus and cuboid to correct and control calcaneal varus. On lateral X‐ray near the Gissane angle, three nails were drilled in a zigzag arrangement perpendicular to the fracture line and the lateral wall of the calcaneus. The three nails were drilled almost in an equilateral triangle or at an angle of 60 degrees to maintain the fixed fractured end. The first nail near the bottom of the calcaneal tubercle was drilled vertically to the fracture line to avoid calcaneal widening. Two Kirschner wires, one high and one low, were fixed in the talus and cuboid at an angle of approximately 45 degrees to prevent height collapse and calcaneal shortening.

## Introduction

As the largest tarsal bone of the foot, the calcaneus bears approximately 50% of the axial pressure load of the human body. The outer layer of the calcaneus is an irregular three‐dimensional structure composed of thin cortical bone surrounding cancellous bone. Due to its unique anatomical structure, there is a great chance of damage. Calcaneal fractures are usually caused by high‐energy trauma, accompanied by varying degrees of skin and soft tissue damage, with clinical manifestations of foot swelling, severe pain, and limited movement, among others. Calcaneal fractures, which account for 2% of all fractures in the whole body, are common tarsal fractures, of which 75% are intra‐articular displaced fractures; 30% of these patients also have calcaneal joint injuries, with a poor prognosis^1^. Displaced intra‐articular calcaneal fractures are absolutely indicated for surgical treatment. The key to the treatment is to restore the level of the articular surface, especially the posterior articular surface, to reduce the incidence of subtalar arthritis, joint stiffness, and restricted internal and external rotation and restore the three‐dimensional structure of the calcaneus, such as the length, width, height, Bohler angle and Gissane angle^2^. The classic L‐shaped lateral extension incision can be used to achieve anatomical reduction of the subtalar joint under direct vision, but it damages the blood supply of local soft tissue in a wide range and can easily lead to postoperative wound infection, skin necrosis and steel plate exposure, with an incidence of complications as high as 18%–25%^3^. The tarsal sinus approach can avoid damaging the blood supply of the foot and reduce the incidence of complications, but its limited field of exposure prevents its use in the treatment of all types of fractures. To reduce surgical complications, percutaneous minimally invasive techniques have been rapidly developed in clinical orthopedics, providing new ideas and methods for the treatment of intra‐articular calcaneal fractures.

Therefore, the purpose of this study was as follows: (i) to evaluate calcaneal forceps in the percutaneous minimally invasive reduction and medial external fixation of Sanders type II and III intra‐articular calcaneal fractures; (ii) to retrospectively compare this minimally invasive reduction and external fixation approach with the tarsal sinus approach in the treatment of Sanders type II and III calcaneal fractures in terms of the curative effect; and (iii) to determine the safety and efficacy of the percutaneous minimally invasive reduction of Sanders type II and III intra‐articular calcaneal fractures with plastic calcaneal forceps combined with medial external fixation. We assume that percutaneous minimally invasive technology can be applied to effectively treat Sanders type II and III intra‐articular calcaneal fractures with other techniques in the future.

## Materials and Methods

### Ethics Approval

This retrospective study was approved by the Ethics Committee of the Affiliated Zhongshan Hospital of Dalian University (No. 6, Jiefang Street, Zhongshan District, Dalian).

### Inclusion and Exclusion Criteria

The inclusion criteria were as follows: (i) diagnosis of closed Sanders type II or III calcaneal fracture on X‐ray or CT examination; (ii) age of 18 to 65 years; (iii) first calcaneal fracture in a single foot; and (iv) complete clinical data and written informed consent from the patient.

The exclusion criteria were as follows: (i) multiple or compound injuries; (ii) open fracture of the lower extremity with nerve and blood vessel injury; (iii) diabetes mellitus or vascular disease affecting the lower extremities; or (iv) pathological calcaneal fracture.

### General Information

Sixty‐four patients with calcaneal fractures admitted to our hospital from January 2010 to January 2016 were retrospectively analyzed and divided into two groups according to the surgical approach, including 30 cases in the plastic calcaneal forceps percutaneous minimally invasive reduction and external fixation group and 34 cases in the minimally invasive tarsal sinus approach group. There was no significant difference between the two groups in age, sex, body mass index, Sanders classification or other general data (*P* > 0.05, Table 1).

**TABLE 1:** Comparison of general data between the two groups

Indicators		MI group (n = 30)	ST group (n = 34)	*t* value	*P* value
Age(year, x ± s)		46.2 ± 3.8	47.3 ± 2.6	0.582	0.621
gender(n)	male female	24 6	29 5	0.058	0.785
BMI(kg/m2, x±s)		25.2 ± 2.8	24.9 ± 3.1	0.283	0.801
Fracture classification(n)	II III	22 8	20 14	0.343	0.692

Each patient's medical history was collected, including age, sex, body mass index, and Sanders classification (Table 1).

### Surgical Technique

**Fig. 1:**
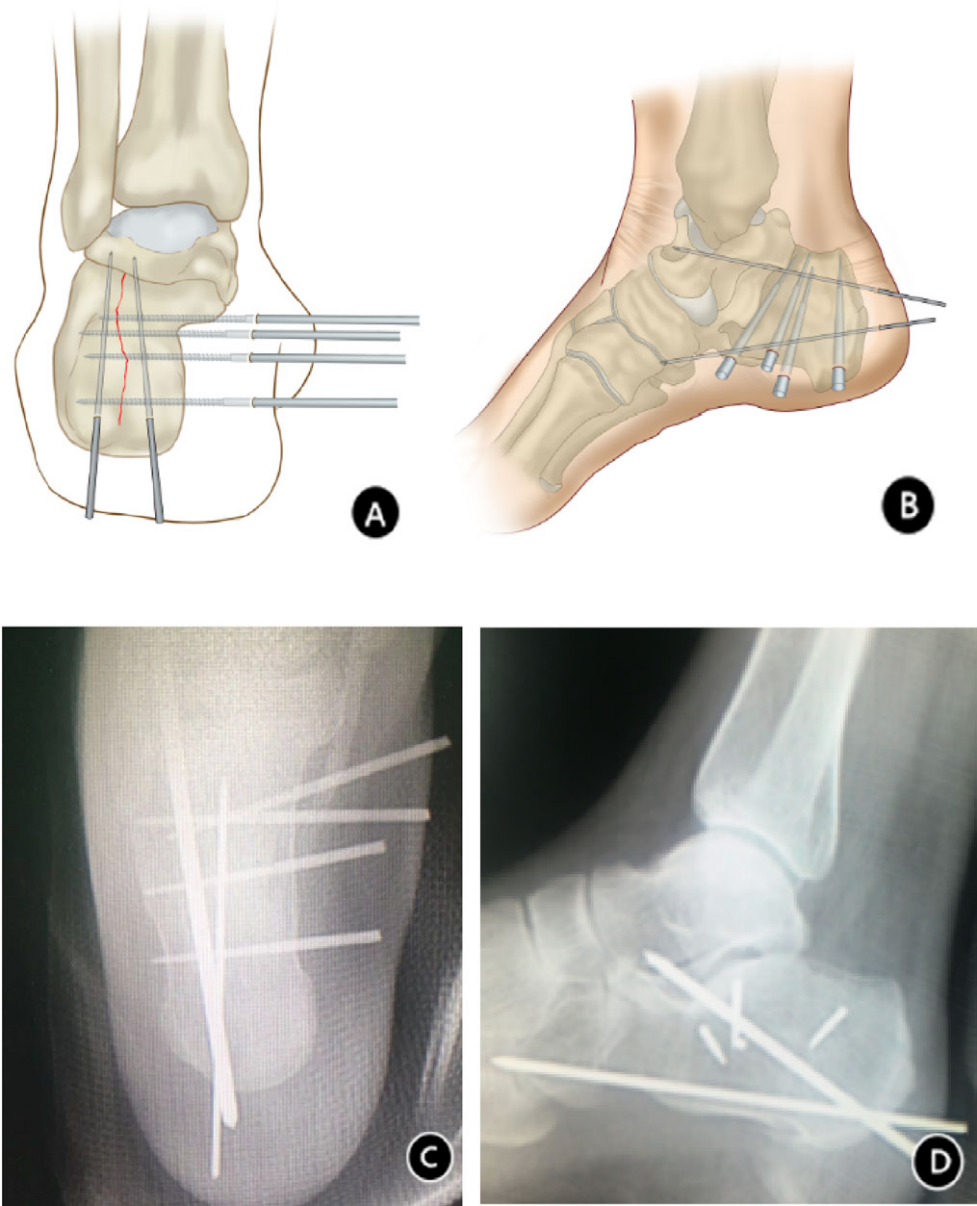
The anatomical structure of the calcaneus was restored by applying traction and extrusion with calcaneus‐shaping forceps. On axial X‐ray, four nails were arranged from the subtalar joint to the calcaneal tuberosity in different planes to provide fracture surface support. Two Kirschner wires drilled from the posterior tuberosity were fixed in the talus and cuboid to correct and control calcaneal varus. On lateral X‐ray near the Gissane angle, three nails were drilled in a zigzag arrangement perpendicular to the fracture line and the lateral wall of the calcaneus. The three nails were drilled almost in an equilateral triangle or at an angle of 60 degrees to maintain the fixed fractured end. The first nail near the bottom of the calcaneal tubercle was drilled vertically to the fracture line to avoid calcaneal widening. Two Kirschner wires, one high and one low, were fixed in the talus and cuboid at an angle of approximately 45 degrees to prevent height collapse and calcaneal shortening.

All patients with intra‐articular calcaneal fractures were treated under epidural anesthesia at our hospital, and the surgery was performed by the same orthopedic surgeon (Dr. Wang Jianchuan). Patients are usually positioned lying on their healthy side with the affected foot on top, and reduction and fixation are performed under C‐arm fluoroscopy. The surgical procedures are described below (Fig. 1).

### Percutaneous Minimally Invasive Medial External Fixation

**Fig. 2:**
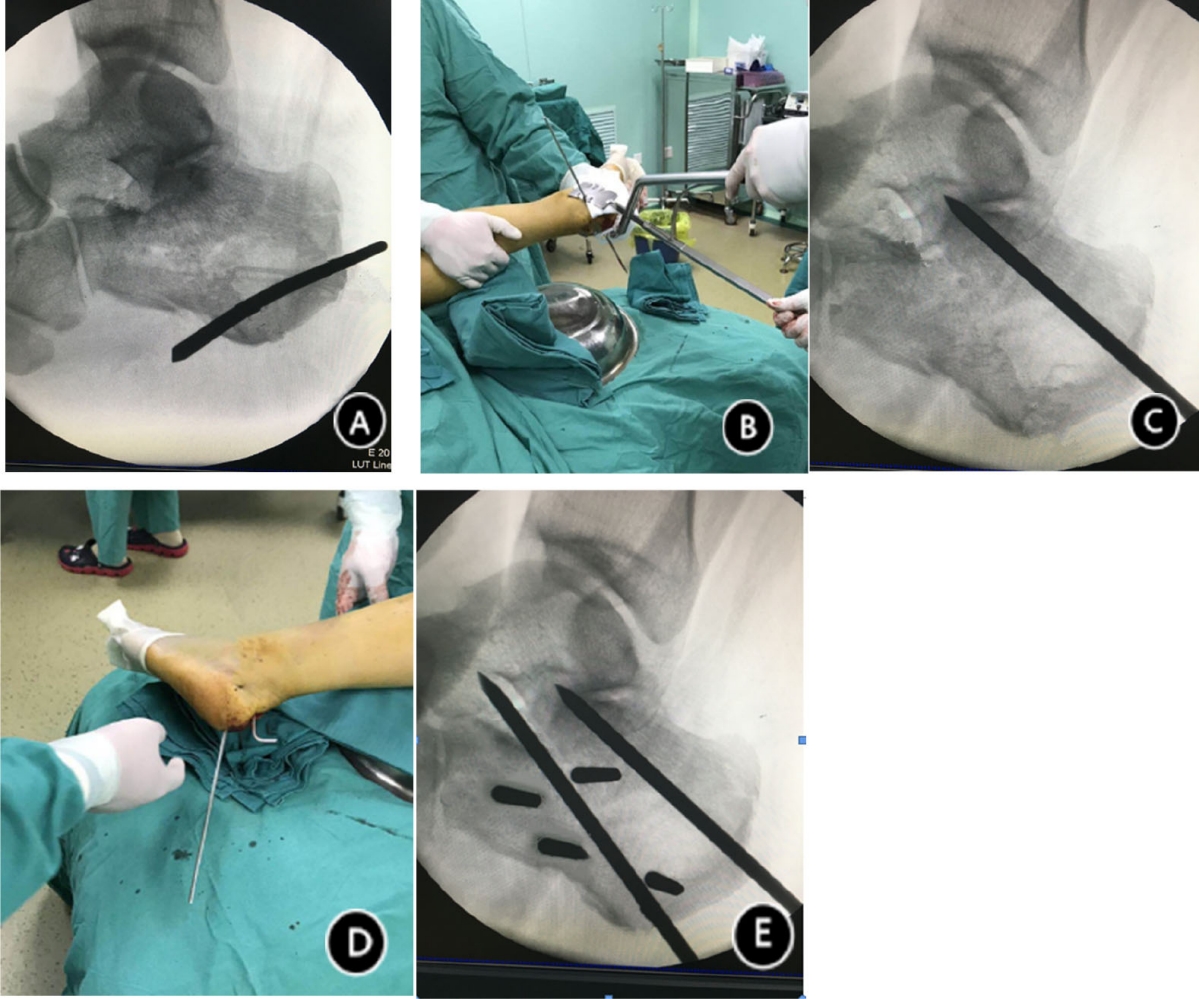
Intraoperative description. (A) A 4.0 wire was drilled into the vertical calcaneal tubercle for traction to restore the length of the calcaneus. (B) The length of the calcaneus was restored under C‐arm fluoroscopy, and reduction forceps were applied to compress the inner and outer walls of the calcaneus and restore the width of the calcaneus, while lateral adjustments were made to correct pronation. (C) When the length, width, and pronation were restored, a 4.0 wire was inserted from the calcaneal tubercle and used to pry the articular surface, restoring the stability and height of the calcaneus. (D) To restore the anatomical stereoscopic structure of the calcaneus and prevent collapse, two Kirschner wires were drilled into the talus. (E) Under C‐arm guidance, 4 nails were inserted into the inner wall for fixation to limit calcaneal width loss and collapse and preserve other anatomical three‐dimensional structures.

The operation was completed by percutaneous reduction with plastic calcaneal forceps. First, the 4.0 wire was drilled vertically into the calcaneus, and the Kirschner wire was placed in the groove of the plastic clamp. Then, the calcaneus was pulled out and inserted into the shortened space to restore the length. At the same time, calcaneal pronation was corrected by traction, and the internal and external calcaneal walls were compressed to restore the width of the calcaneus; then, the Kirschner wire was drilled into the calcaneal tuberculum from the rear. Under C‐arm guidance, the collapsed articular surface was reduced by prying and removal, and the calcaneal height was restored by fixing the Kirschner wire to the talus. The length, width and height of the calcaneus were restored under fluoroscopy; the lower articular surface was flat, and the internal fixation was moderate. According to the fracture line, 3‐4 headless compression screws were drilled into the fractured end in a three‐dimensional shape. Headless pressure screws with a self‐tapping function can be applied for the pressure fixation of fractures. Finally, fluoroscopy was performed to confirm the location of the fracture and internal fixation instrumentation (Fig. 2).

### Minimally Invasive Surgery with the Tarsal Sinus Approach

In the tarsal sinus group, the surgical incision extended 1 cm from the tip of the lateral malleolus forward to the base of the fourth metatarsal bone, parallel to the plantar fascia, with a length of approximately 4‐5 cm. The skin and fascia were incised to expose the calcaneal insertion of the sural nerve, the peroneal tendon and the calcaneal ligament. The subtalar joint was exposed by sharp dissection. A Kirschner wire was drilled into the posterior calcaneal body, and the collapsed articular surface was restored under direct vision with the pry technique. The width of the calcaneus was restored, and pronation deformity was corrected with a Kirschner wire for anti‐traction and manual lateral extrusion. The Kirschner wire was temporarily fixed, and the reduction effect was confirmed by fluoroscopy. Appropriate steel plates were selected, the periosteum was stripped, and the steel plates were inserted under the skin over the calcaneus. The fixation screws were drilled through the small percutaneous incision, incision drainage was established, and the incision was sutured.

### Postoperative Treatment

Routine antibiotics were used 3 days after the operation, and low‐molecular‐weight heparin was given until 35 days after the operation. The affected foot was raised, the lower leg was fixed with plaster, and the root of the inner headless pressurized screw was disinfected with sterile alcohol daily to prevent nail tract infection. Activities of the hip and knee were allowed immediately after the operation. The patient was allowed to walk with two crutches on the second day after the surgery, but the patient was required not to carry weight on the affected ankle. The headless pressure screw thread was removed at 6 weeks after the surgery. Partial weight bearing was allowed after 6 weeks, and full weight bearing was allowed after 12 weeks.

### Outcomes

**Fig. 3:**
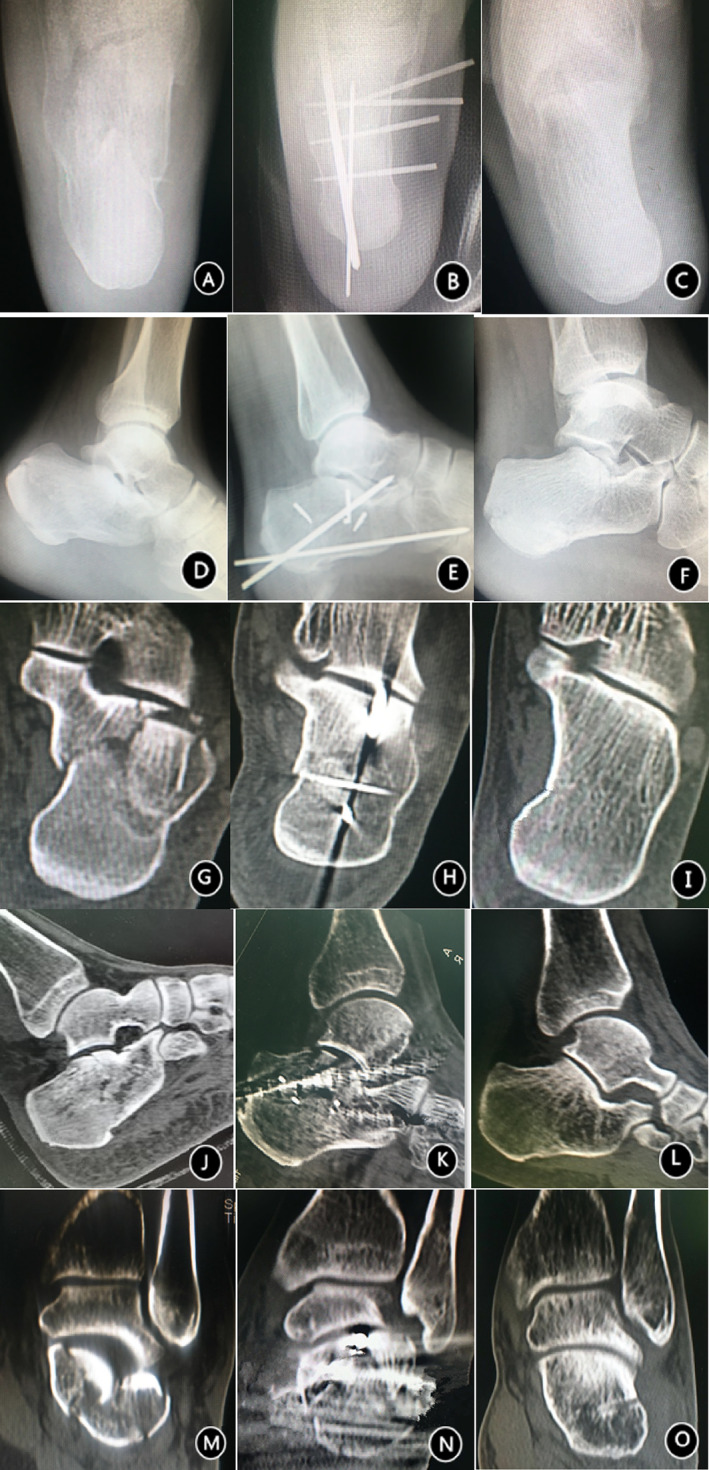
Patients were followed up with minimally invasive techniques. A 38‐year‐old man with a comminuted Sanders type IIIAB left calcaneal fracture caused by falling off a ladder was treated with orthopedic forceps and minimally invasive reduction with percutaneous headless compression screw internal fixation. The final follow‐up was at 33.2 months. (A) Axial X‐ray showing that the calcaneus was shortened and widened, with angled inversion. (B) After reduction, the calcaneal length, width, and inversion were corrected, and the headless compression screw on the inside was fixed and stable. (C) Good recovery of the calcaneus at the last follow‐up. (D) Lateral X‐ray showing collapse of the articular surface, a decrease in the Bohler angle such that it became negative, and a decrease in the height of the calcaneus. (E) Postoperative X‐ray showing recovery of the calcaneal height, return of the Bohler angle to within the normal range, and reliable headless compression screw fixation. (F) Normal calcaneal morphology at the last follow‐up. (G) Coronal CT showing calcaneal inversion and shortening, significantly calcaneal widening, and collapse and displacement of the articular surface. (H) Percutaneous reduction using orthopedic forceps restored the length of the calcaneus, corrected the inversion, and restored the articular surface. (I) At the last follow‐up, there was no loss of calcaneal length or varus, and the articular surface was flat. (J) Preoperative sagittal CT showing collapse and compression of the posterior calcaneal articular surface. (K) Prying with a Kirschner wire to restore the posterior calcaneal articular surface. (L) Flattening of the posterior calcaneal articular surface at the last follow‐up. (M) Preoperative CT showing displacement of the subtalar articular surface. (N) Smooth restoration of the subtalar articular surface and headless compression screw fixation. (O) Stable subtalar articular surface at the last follow‐up, with no traumatic arthritis.

**Fig. 4:**
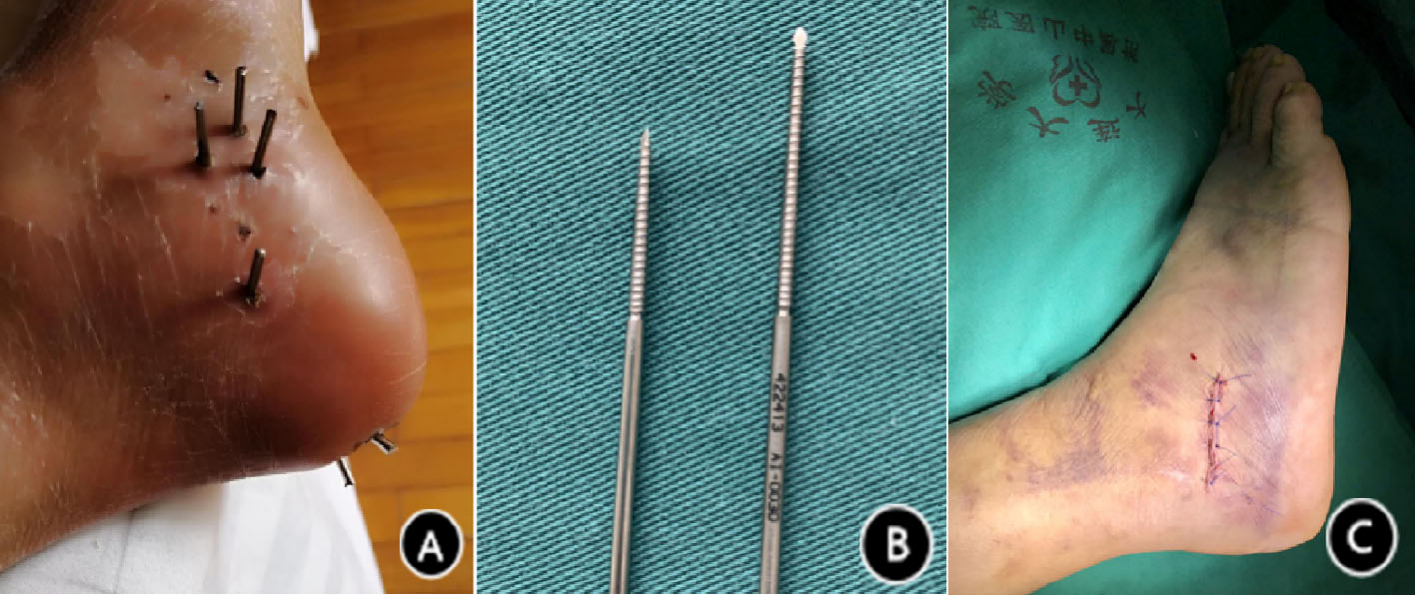
(A) Appearance of inner headless pressure screws after percutaneous minimally invasive reduction and external fixation. The screws can be retained 1 cm from the surface of the skin to facilitate nursing and reduce displacement and loosening caused by collision with external objects. The three headless pressure screws were fixed in a certain shape. (B**)** Headless pressure screws, with a self‐tapping pressure function, stabilizes the fracture very well. (C**)** Appearance of the incision after the minimally invasive tarsal sinus approach.

Clinical data were collected and measured preoperatively, intraoperatively, 1 day, 4 weeks, 8 weeks, 12 weeks, 6 months, and 12 months postoperatively and at the final follow‐up (2 years or more) (Figs. 3 and 4).

### Patient follow‐up after treatment with the tarsal sinus approach

A 40‐year‐old, female with a right heel bone Sanders type IIA comminuted fracture underwent treatment with the minimally invasive tarsal sinus approach with open reduction and plate fixation; the final follow‐up was at 31.8 months. (A) Lateral X‐ray showing a decrease in the calcaneal height and a decrease in the Bohler angle and Gissane angle. (B) Postoperative X‐ray showing restoration of the calcaneal height, restoration of the Bohler and Gissane angles to within the normal range, and good internal fixation positioning. (C) Lateral calcaneal X‐ray at the final follow‐up. (D) Axial X‐ray showing a reduced calcaneal length, an increased calcaneal width, and increased calcaneal varus. (E) Restoration of the length and width of the heel bone, correction of calcaneal varus, and stabilization of the plate position. (F) Axial X‐ray of the calcaneus at the final follow‐up. (G) Coronal CT showing a reduced calcaneal length, an increased calcaneal width, and increased calcaneal varus. (H) Restoration of the length and width of the calcaneus and correction of calcaneal varus. (I) At the last follow‐up, there was no loss of correction of the length, width or varus of the calcaneus, and the joint surface was flat.

#### Functional Scores

Ankle function was evaluated according to the American Orthopedic Foot and Ankle Society (AOFAS) Ankle and Hindfoot score and the Maryland Ankle Function score^4^.

#### American Orthopedic Foot and Ankle Society (AOFAS) Ankle and Hindfoot Score

Postoperative function was evaluated according to the American Orthopedic Foot and Ankle Society (AOFAS) Ankle and Hindfoot score. The score includes items on pain, function, and alignment, with a maximum score of 100. A score of 90–100 indicates an excellent outcome; 80–89, good; 70–79, fair; and < 70, poor.

#### Visual Analogue Scale Score

The visual analogue scale (VAS) is used to measure pain intensity. The VAS score ranges from 0–10, with a lower score indicating less pain and a higher score indicating more pain^5^.

#### Short Health Questionnaire (SF‐36)

The SF‐36 Health Status Questionnaire^6^ was used to evaluate the clinical efficacy of treatment. The higher the score was, the higher the quality of life of the respondents.

#### Maryland Foot Function Score

The Maryland Foot Function Score^7^ is used to assess pain and function, with an overall score of 100. A score of 90–100 indicates an excellent result; 75–89, good; 50–74, fair; and <50, poor).

#### Radiological Assessment

Clinical, X‐ray and CT follow‐up examinations were performed at 1 day, 4 weeks, 8 weeks, 12 weeks, 6 months, and 12 months after surgery and every year thereafter. The calcaneal length, width, and height, Bohler angle, Gissane angle, and calcaneal varus angle were measured before and after surgery and at the last follow‐up.

### Statistical Analysis

SPSS 23.0 software was used for statistical analysis. Measurement data are expressed as the mean ± standard deviation (x ± s). Independent sample T tests were used for intergroup comparisons, and paired T tests were used for intragroup comparisons. Enumeration data are presented as a rate (%) and were compared by the chi‐square test. The test level was 0.05.

## Results

### General Results

From January 2010 to January 2016, 64 patients with intra‐articular calcaneal fractures were admitted, including 30 patients in the percutaneous minimally invasive reduction and medial external fixation group and 34 patients in the tarsal sinus approach group. Patients in both groups underwent follow‐up, with an average follow‐up of 24.8 months (range, 12–50 months). The age in the minimally invasive percutaneous medial external fixation group was 46.2 ± 3.8 years, and the age in the minimally invasive tarsal sinus group was 47.3 ± 2.6 years. The BMI was 25.2 ± 2.8 kg/m2 in the minimally invasive percutaneous medial external fixation group and 24.9 ± 3.1 kg/m2 in the minimally invasive tarsal sinus group. Percutaneous minimally invasive medial external fixation was performed in 22 cases of Sanders type II fracture and eight cases of Sanders type III fractures. Minimally invasive tarsal sinus surgery was performed in 20 cases of Sanders type II fracture and 14 cases of Sanders type III fracture.

### Comparison of Operative Indexes

The time to surgery, hospital stay, intraoperative blood loss, and operation time were significantly lower in the percutaneous minimally invasive medial external fixation group than in the tarsal sinus group (*P* < 0.01). Regarding postoperative complications, there were two cases of internal fixation loosening in the minimally invasive group and three cases of wound infection and three cases of gastritis nerve injury in the tarsal sinus group. The incidence of complications was significantly lower in the minimally invasive group than in the tarsal sinus group (*P* < 0.01) (Table 2).

**TABLE 2:** Comparison of surgical indexes between the two groups

Indicators	MI group (n = 30)	ST group (n = 34)	*t* value	*P* value
Waiting time for surgery (d)	3.65 ± 0.76	7.27 ± 0.98	10.231	<0.001
Length of stay (min)	8.73 ± 2.61	12.65 ± 1.86	14.624	<0.001
Intraoperative Blood loss (ml)	50.52 ± 8.23	98.83 ± 15.65	13.755	<0.001
operation time (min)	59.63 ± 9.97	76.27 ± 8.87	5.547	<0.001
Complications (case)	2	6	3.213	<0.001

#### Imaging Results at the Last Follow‐up in Both Groups

The length, width, and height of the calcaneus were significantly increased in the 64 patients at the last follow‐up compared with before surgery, and the width was significantly decreased after comparison with before surgery in both groups (*P* < 0.01) (Table 3).

**TABLE 3:** Comparison of imaging follow‐up measurements between the two groups

Indicators(mm)	Measuring time	MI group (n = 30)	ST group (n = 34)	*t* value	*P* value
Calcaneal length	preoperative	67.21 ± 1.88	66.69 ± 2.18	0.968	0.534
	Last follow‐up	72.21 ± 2.16	71.62 ± 1.35	0.898	0.645
	*t* value	8.184	7.852		
	*P* value	<0.001	<0.001		
Calcaneal width	preoperative	38.44 ± 1.32	37.74 ± 2.43	0.341	0.276
	Last follow‐up	33.21 ± 1.45	35.34 ± 3.37	0.7672	0.264
	*t* value	6.223	7.634		
	*P* value	<0.001	<0.001		
Calcaneal height	preoperative	45.87 ± 2.43	44.95 ± 1.87	0.075	0.243
	Last follow‐up	48.45 ± 1.13	47.66 ± 1.76	0.235	0.154
	*t* value	9.586	7.855		
	*P* value	<0.001	<0.001		

The Bohler angle and Gissane angle of the calcaneus were significantly increased before the operation and significantly decreased at the last follow‐up, and the change in calcaneal varus angle was significant in the minimally invasive group (*P* < 0.01). At corresponding time points, there were no statistically significant differences in the calcaneal length, width, or height, Bohler angle, Gissane angle, or calcaneal varus angle between the two groups (*P* > 0.05) (Table 4).

**TABLE 4:** Comparison of imaging follow‐up measurements between the two groups

Indicators (°)	Measuring time	MI group (n = 30)	ST group (n = 34)	*t* value	*P* value
Bohler angle	preoperative	15.64 ± 1.32	16.69 ± 0.98	1.023	0.125
	follow‐up	32.13 ± 2.34	33.52 ± 1.05	1.114	0.168
	*t* value	85.684	88.643		
	*P* value	<0.001	<0.001		
Gissane angle	preoperative	98.62 ± 3.21	96.74 ± 2.43	1.331	0.194
	follow‐up	128.2 ± 2.45	126.54 ± 3.37	1.622	0.208
	*t* value	24.857	20.634		
	*P* value	<0.001	<0.001		
Varus angle	preoperative	12.21 ± 2.21	12.06 ± 2.15	0.178	0.743
	follow‐up	1.85 ± 1.13	2.56 ± 1.46	0.345	0.134
	*t* value	7.213	4.45 ± 1.18		
	*P* value	<0.001	<0.001		

### Clinical Function Scores of Patients in Both Groups at the Last Follow‐up

The AOFA score, VAS score, SF‐36 score and Maryland score were determined in 64 patients before surgery and at the last follow‐up (Table 5). There were statistically significant differences in the VAS score, SF‐36 score and Maryland score between the groups before and after the operation (*P* < 0.05). There were statistically significant differences in the VAS, SF‐36, and Maryland scores between before and after the operation (*P* < 0.05).

**TABLE 5:** Clinical efficacy of minimally invasive approach in the two groups at the last follow‐up

Group	AOFAS score (score, x ± s)	VAS score (score, x ± s)	SF‐36score (score, x ± s)	Maryland score (score, x ± s)
MI group(30)	85.28 ± 8.21	0.84 ± 1.21	82.95 ± 3.25	83.56 ± 3.32
ST group(34)	83.32 ± 7.69	1.85 ± 1.32	80.71 ± 5.42	81.85 ± 2.41
*t* value	0.78	0.52	1.51	0.66
*P* value	0.61	0.43	0.09	0.49

#### AOFAS Score

The AOFAS score in the minimally invasive medial external percutaneous fixation group was 85.28 ± 8.21 at the last follow‐up, and the AOFAS score in the minimally invasive tarsus group was 83.32 ± 7.69 at the last follow‐up.

#### VAS Score

The VAS score in the minimally invasive medial external percutaneous fixation group was 0.84 ± 1.21 at the last follow‐up, and the VAS score in the minimally invasive tarsal sinus group was 1.85 ± 1.32 at the last follow‐up.

#### SF‐36 Score

The SF‐36 score in the minimally invasive medial external percutaneous fixation group was 82.95 ± 3.25 at the last follow‐up, and the SF‐36 score in the minimally invasive tarsal sinus group was 80.71 ± 5.42 at the last follow‐up.

#### Maryland Score

At the last follow‐up, the Maryland score was 83.56 ± 3.32 in the percutaneous minimally invasive medial external fixation group and 81.85 ± 2.41 in the minimally invasive tarsal sinus group.

### Analysis

#### Age

The mean age at surgery was 46.2 ± 3.8 years in the minimally invasive percutaneous medial external fixation group and 47.3 ± 2.6 years in the minimally invasive tarsal sinus group. The independent sample T test showed no significant correlation between age and functional outcomes (*P* = 0.621).

#### Sex

Twenty‐four males and six females were included in the percutaneous minimally invasive medial external fixation group. There were 25 males and nine females in the minimally invasive tarsal sinus group. The independent sample T test showed no significant correlation between sex and functional results (*P* = 0.785).

#### Body Mass Index (BMI)

The BMI was 25.2 ± 2.8 kg/m2 in the minimally invasive percutaneous medial external fixation group and 24.9 ± 3.1 kg/m2 in the minimally invasive tarsal sinus group. The independent sample T test showed no significant correlation between BMI and functional outcomes (*P* = 0.801).

#### Fracture Classification

Percutaneous minimally invasive external fixation was performed for 22 cases of Sanders type II fracture and eight cases of Sanders type III fracture. Minimally invasive tarsal sinus surgery was performed for 20 cases of Sanders type II fracture and 14 cases of Sanders type III fracture. No significant correlation was found between fracture classification and functional outcome by independent sample T test (*P* = 0.692).

### Imaging Measurement Analysis

#### Calcaneal Length

In the minimally invasive percutaneous medial external fixation group, the length of the calcaneus was 67.21 ± 1.88 mm before surgery and 72.21 ± 2.16 mm at the last follow‐up. In the minimally invasive tarsal sinus group, the calcaneal length was 66.69 ± 2.18 mm before surgery and 71.62 ± 1.35 mm at the last follow‐up, showing a statistically significant difference (*P* = 0.00085), while there was no statistically significant difference between the two groups (*P* = 0.589).

#### Calcaneal Width

The width of the calcaneus in the minimally invasive percutaneous medial external fixation group was 38.44 ± 1.32 mm before surgery and 33.21 ± 1.45 mm at the last follow‐up. The width of the calcaneus was in the minimally invasive tarsal sinus group 37.74 ± 2.43 mm before surgery and 35.34 ± 3.37 mm at the last follow‐up. The differences between the two time points were statistically significant (*P* = 0.00033). There was no significant difference between the two groups (*P* = 0.271).

#### Calcaneal Height

In the minimally invasive group, the height of the calcaneus was 45.87 ± 2.43 mm before surgery and 48.45 ± 1.13 mm at the last follow‐up. In the minimally invasive tarsal sinus group, the height of the calcaneus was 44.95 ± 1.87 mm before surgery and 47.66 ± 1.76 mm at the last follow‐up. The difference between the two time points was statistically significant (*P* = 0.00027). There was no significant difference between the two groups (*P* = 0.198).

#### Bohler Angle

In the percutaneous minimally invasive external fixation group, the Bohler angle was 15.64 ± 1.32° before surgery and 32.13 ± 2.34° at the last follow‐up. In the minimally invasive tarsal sinus group, the Bohler angle was 16.69 ± 0.98° before surgery and 33.52 ± 1.05° at the last follow‐up, with a significant difference between the two time points (*P* = 0.00068). There was no statistically significant difference between the two groups (*P* = 0.146).

#### Gissane Angle

In the percutaneous minimally invasive external fixation group, the Gissane angle was 98.62 ± 3.21° before surgery and 128.2 ± 2.45° at the last follow‐up. In the minimally invasive tarsal sinus group, the Gissane angle was 96.74 ± 2.43° before surgery and 126.54 ± 3.37° at the last follow‐up, with a significant difference between the two time points (*P* = 0.00077). There was no statistically significant difference between the two groups (*P* = 0.201).

#### Varus Angle

In the percutaneous minimally invasive external fixation group, the varus angle was 12.21 ± 2.21° before surgery and 1.85 ± 1.13° at the last follow‐up. In the minimally invasive tarsal sinus group, the varus angle was 12.06 ± 2.15° before surgery and 2.56 ± 1.46° at the last follow‐up, with a significant difference between the two time points (*P* = 0.00061). There was no statistically significant difference between the two groups (*P* = 0.438).

This study included all cases of Sanders type II and III calcaneal fractures, preoperatively excluding cases of Sanders type IV fracture, and patients with a history of ankle surgery and with vascular disease affecting the lower limbs. We examined the clinical features of these patients, including age, sex, fracture type, and operation method, and found no significant correlation with the outcomes.

## Discussion

The calcaneus is the most commonly fractured tarsal bone, accounting for approximately 60% of all tarsal fractures and 2% of all fractures throughout the body^8^. Approximately 75% of calcaneal fractures occur in the joint. Displaced calcaneal fractures are disabling injuries that mostly occur in young and active manual workers. This kind of injury undoubtedly has a high economic impact on both the family and society. The ideal treatment for displaced intra‐articular calcaneus fractures is still controversial. Because of the complex anatomical structure of the calcaneus, fractures often involve the subtalar joint, so the treatment of calcaneal fractures is a challenging clinical problem for orthopedic trauma surgeons^9^. With the gold standard lateral L‐incision approach, the wall can be fully exposed, the heel bone can be manipulated under the joint surface, and the bone can be examined after being reset; additionally, this method also provides sufficient fixation strength. However, these advantages are obtained at the expense of the skin around the incision, with destruction of the blood supply of the external wall, and the rate of incision‐related complications is as high as 30%^10^.

To reduce the incidence of surgical complications and soft tissue damage, especially in patients with multiple systemic complications or a high surgical risk, many minimally invasive techniques have been developed and applied^11^. Minimally invasive techniques for the treatment of calcaneal fractures can effectively reduce surgical complications, shorten the fracture healing time, and thus improve the quality of life of patients^12^. At present, commonly used minimally invasive techniques include percutaneous pry removal with external plaster fixation, percutaneous external fixation, percutaneous balloon dilatation reduction with bone cementation, and tarsal sinus plate internal fixation combined with percutaneous nail fixation^13^. The minimally invasive tarsal sinus incision was applied by Ebraheim in the clinical treatment of calcaneal fractures in 2000^14^. This approach is located on the dorsalis of the foot, which reduces the chance of damage to the lateral calcaneal artery, reduces the amount of soft tissue dissection, and has little impact on the blood supply of the flap. Wang *et al*.^15^ treated 18 cases of calcaneal fracture by the tarsal sinus approach and found no incision complications. Mostafa *et al*.^16^ also reported the treatment of 18 cases of calcaneal fracture with the tarsal sinus approach. The average Bohler angle was corrected from 5.1° preoperatively to 34.6° postoperatively, with the correction rate reaching 91.4%; the rate of heel height correction was 95.2%; the incidence of arthritis was 27.8%; and the score was 77.8%. Due to the limited incision of the tarsal sinus approach, the exposure scope is limited, and the posterior and lateral sides of the calcaneus cannot be fully exposed as with the lateral L incision, which makes it more difficult to restore the height and width of the calcaneus during the operation and makes it easy to separate soft tissues and remove the damaged femoral nerve^17^.

Percutaneous closed reduction and external fixation mainly involves manual reduction or the use of Kirschner wires, external fixators, point‐type reduction forceps and other auxiliary instruments to pry or pull the fractured bone. These techniques are widely used in clinical practice. Percutaneous fixation is feasible for any type of intra‐articular calcaneal fracture, especially for patients with severe soft tissue injury^18^. External fixation for patients with mild soft tissue injury is restricted in terms of the operation time and fracture type but is advantageous in terms of wound care and can not only maximize functional recovery of the heel bone but also effectively reduce the incidence of complications. Furthermore, the operative technology is simple, the degree of damage is low, and the method can restore the heel bone length, width and height as well as the Bohler angle and Gissane angle, allowing early activity and partial weight bearing^19^. Early loading can promote the formation of the subtalar joint and slow down the occurrence of traumatic arthritis. As external fixation technology is not hindered by soft tissue injury or limb swelling, surgery can be performed early. Studies have shown that the time to surgery is an important factor affecting the surgical outcomes; surgery should be performed within 3 to 5 days, especially in the case of percutaneous or minimally invasive surgery^20^. Pezzoni *et al*.^21^ reported similar results for traditional Essex Lopresti prying reduction in the modified “breyashi bridge” technique, which can achieve a satisfactory Bohler angle under fluoroscopic percutaneous reduction and provide stable fixation with only 3–4 Kirschner wires. Stulik *et al*.^22^ treated 247 patients with calcaneal fractures with pry reduction and Kirschner wire fixation. Among all 278 patients, 1% showed skin necrosis, 4.5% showed redisplacement, 73.9% achieved anatomical reduction, and only 8.7% showed nail tract infection. Therefore, regardless of the kind of surgery adopted, it is crucial to restore the anatomical appearance of the calcaneus first. The standard of reduction should not be lowered because of the choice of minimally invasive surgery. To achieve the therapeutic goal, the anatomical structure of the calcaneus should be restored first, and then strong fixation should be provided to maintain reduction. However, the premise is the strong fixation of both the inside and outside walls of the heel, but in practice, the lateral calcaneal wall is thin and easily comminuted. It is difficult to obtain sufficiently powerful control points for screws in this location, and fragment fixation can be influenced by biomechanics, the trabecular bone, the bone density distribution, and the characteristics of the joint surface. The calcaneal tubercle and inner calcaneal inside wall are below three areas with the highest bone density, while the cortical bone is the densest^23^. Therefore, we placed headless compression screws in the medial wall to maintain stable reduction. We used calcaneal forceps combined with medial compression screws for calcaneal fracture fixation. There were no cases of soft tissue complications or nail tract infection. When a calcaneal fracture occurs, there will be subcutaneous swelling and blood stasis in the calcaneal bone, and a long period of time will be required to reduce the swelling. Performing surgery too early could cause skin infection or necrosis, internal fixation leakage and other complications. This technique is advantageous in that it can be used in patients with acute edema and soft tissue injury, greatly shortening the operation and hospital stay and reducing the risk of surgical complications.

Rammelt *et al*.^24^ also believed that the earlier the operation was, the better the percutaneous anatomical reduction would be because a scar would form around the fracture over time, increasing the difficulty of reduction. Percutaneous fixation can be performed early without the need to wait for the swelling to subside, thus avoiding surgical delay and reducing the difficulty of reduction. Percutaneous pry removal is easy to perform, reduces the damage to soft tissue around the calcaneus caused by open surgery and greatly reduces the risk of wound‐related necrosis and infection, especially in patients with a long‐term smoking history, drug addiction, diabetes, peripheral vascular disease and open fractures. The most important thing is that percutaneous lateral wall external fixation is equivalent to conservative treatment, and the whole process is minimally invasive, which is more acceptable to patients.

### Limitations

This study has some limitations: (i) the sample size is relatively small, so more samples need to be included in future work; (ii) in a radiological assessment by a single surveyor, there may be measurement error; and (iii) the average postoperative follow‐up duration in this study was more than 2 years, which is relatively short. Thus, more mid‐ and long‐term follow‐up studies are required.

### Conclusion

The results of this study show that plastic calcaneal forceps combined with minimally invasive reduction and medial external fixation was equally as effective as the tarsal sinus approach in the treatment of Sanders type II and III calcaneal fractures and was an effective and reliable alternative treatment.

Assuming that good clinical efficacy on long‐term follow‐up, this approach will undoubtedly serve as a new treatment technique for intra‐articular calcaneal fractures. (i) The whole operation follows a percutaneous minimally invasive technique, with no need to cut and peel soft tissue, and is not prone to the risk of skin necrosis or external leakage as is internal fixation. (ii) This surgery is equivalent to conservative treatment, which is easy for patients to accept, and the incidence of deformity, traumatic arthritis and bone loss is low. (iii) There is no need to worry about the presence of diabetes, smoking or lower extremity vascular diseases in patients, and internal fixation can be removed in the outpatient clinic, which reduces the patient's economic burden. (iv) The distance between the threads of the headless compression screws gradually increases from the back end to the front end, which increases the speed at which the screw can be inserted into the bone and gradually compresses the bone fragments.

## Author Contributions

Wang Jianchuan was responsible for the manuscript writing and article conception; Liu Jibin was responsible for statistical analysis; and Wang was responsible for case and data collection.

## References

[1] Simon P , Goldzak M , Eschler A , *et al*. Reduction and internal fixation of displaced intra‐articular calcaneal frac‐tures with a locking nail:a prospective study of sixty nine cases. Int Orthop, 2015, 39: 2061–2067. 26152240 10.1007/s00264-015-2816-5

[2] Kline AJ , Anderson RB , Davis WH , *et al*. Minimally invasive technique versus an extensile lateral approach for intra articular ealcaneal fractures. Foot Ankle Int, 2013, 34: 773–780. 23460669 10.1177/1071100713477607

[3] Backes M , Schepers T , Beerekamp MS , *et al*. Wound infections following open reduction and internal fixation of calcaneal fractures with an extended lateral approach. Int Orthop, 2014, 4: 767–773. 10.1007/s00264-013-2181-1 PMC3971279 24281853

[4] Kitaoka HB , Alexander IJ , Adelaar RS , *et al*. Clinical rating systems for the ankle—hindfoot, midfoot, hallux, and lesser toes. Foot Ankle Int, 1997, 18: 187–188. 28799420 10.1177/107110079701800315

[5] Hildebrand KA , Buckley RE , Mohtadi NG , Faris P . Functional outcome measures after displaced intra‐articular calcaneal fractures. J Bone Joint Surg Br, 1996, 78: 119–123. 8898141

[6] McDowell I , Newell C . Measuring Health: A guide to rating scales and questionnaires. Journal of Nutrition Education, 1996, 20: 1988. 10.1016/S0022-3182(88)80070-0.

[7] Sanders R , Fortin P , DiPasquale T , *et al*. Operative treatment in 120 displaced intraarticular calcaneal fractures. Results using a prognostic computed tomography scan classification. Clin Orthop Relat Res, 1993, 290: 87–95. 8472475

[8] Yao H , Liang TZ , Xu Y , *et al*. Sinus tarsi approach versus extensil e lateral approach for displaced intra‐ articular calcaneal fracture: a meta‐analysis of current evidence base. J Orthop Surg Res, 2017, 12: 43–44. 28288661 10.1186/s13018-017-0545-8 PMC5348794

[9] Guerado E , Bertrand ML , Cano JR . Management of calcaneal fractures: what have we learnt over the years? Injury, 2012, 43: 1640–1650. 22664393 10.1016/j.injury.2012.05.011

[10] Backes M , Schep NW , Luitse JS , *et al*. The effect of postoperative wound infections on functional outcome following intra‐articular calcaneal fractures. Arch Orthop Trauma Surg, 2015, 135: 1045–1052. 25913906 10.1007/s00402-015-2219-5 PMC4513207

[11] Van Hoeve S , Poeze M . Outcome of minimally invasive open and percutaneous techniques for repair of calcaneal fractures: a systematic review. J Foot Ankle Surg, 2016, 55: 1256–1263. 27555351 10.1053/j.jfas.2016.07.003

[12] Zhang F , Tian H , Li S , *et al*. Meta‐analysis of two surgical approaches for calcaneal fractures:sinus tarsi versus extensile lateral approach. ANZ J Surg, 2017, 87: 126–131. 28122417 10.1111/ans.13869

[13] Feng Y , Shui X , Wang J , *et al*. Comparison of percutaneous can‐nulated screw fixation and calcium sulfate cement grafting ver‐sus minimally invasive sinus tarsi approach and plate fixation for displaced intra‐articular calcaneal fractures:a prospective randomized controlled trial. BMC Musculoskelet Disord, 2016, 17: 288. 27422705 10.1186/s12891-016-1122-8 PMC4946135

[14] Hospodar P , Guzman C , Johnson P , *et al*. Treatment of displaced calcaneus fractures using a minimally invasive sinus tarsi approach. Orthopedics, 2008, 11: 11–12. 10.3928/01477447-20081101-08 19226087

[15] Wang Z , Wang XH , Li SL , *et al*. Minimally invasive(sinus tarsi) approach for calcaneal fractures. J Orthop Surg Res, 2016, 11: 164. 10.1186/s13018-016-0497-4. 28010733 PMC5180402

[16] Mostafa MF , El‐Adl G , Hassanin EY , *et al*. Surgical treatment of displaced intra‐articular calcaneal fracture using a single small lateral approach. Strategies Trauma Limb Reconstr, 2010, 5: 87–95. 21811904 10.1007/s11751-010-0082-z PMC2918739

[17] Veltman ES , Doornberg JN , Stufkens SA , *et al*. Long term outcomes of 1,730 calcaneal fractures: systematic review of the literature. J Foot Ankle Surg, 2013, 52: 486–490. 23663876 10.1053/j.jfas.2013.04.002

[18] Besch L , Waldschmidt JS , Daniels‐Wredenhagen M , *et al*. The treatment of intra‐articular calcaneus fractures with severe soft tis‐sue damage with a hinged external fixator or internal stabilization:long‐term results. J Foot Ankle Surg, 2010, 49: 8–15. 20123280 10.1053/j.jfas.2009.07.019

[19] Sengodan VC , Sengodan MM . Early weight‐bearing using percutaneous external fixator for calcaneal fracture. J Surg Tech Case Rep, 2012, 2: 98–102. 10.4103/2006-8808.110263 PMC3673370 23741585

[20] Tornetta P III . Percutaneous treatment of calcaneal fractures. Clin Orthop Relat Res, 2000, 375: 91–96. 10.1097/00003086-200006000-00011 10853157

[21] Pezzoni M , Salvi AE , Tassi M , *et al*. A minimally in vasive reduction and synthesis method. For calcaneal fracture: the “Brixian bridge” technique. J Foot Ankle Surg, 2009, 48: 85–88. 19110166 10.1053/j.jfas.2008.10.008

[22] Stulik J , Stehlik J , Rysavy M , *et al*. Minimally‐invasive treatment of intra‐articular fractures of the calcaneum. Bone Joint Surg Br, 2006, 88: 1634–1641. 10.1302/0301-620X.88B12.17379 17159178

[23] Mahato NK . Morphology of sustentaculum tali: biomechanical importance and correlation with angular dimensions of the talus. Foot (Edinb), 2011, 21: 179–183. 21855319 10.1016/j.foot.2011.06.001

[24] Rammelt S , Amlang M , Zwipp H , *et al*. Percutaneous treatment of less severe intraarticular calcaneal fractures. Clin Orthop, 2010, 468: 983–990. 19582524 10.1007/s11999-009-0964-x PMC2835587

